# Happy-Productive Patterns in Education: Why Well-Being and Performance Valences Matter

**DOI:** 10.17505/jpor.2026.29455

**Published:** 2026-08-24

**Authors:** Amalia R. Pérez-Nebra, Fabiana Queiroga, Núria Tordera, Marta Gil-Lacruz

**Affiliations:** 1 Bienestar y Capital Social (BYCS), Universidad de Zaragoza, Psychology and Sociology Department, Zaragoza, Spain. Email: amaliaraquel.perez@unizar.es https://orcid.org/0000-0001-8386-1233; 2 Laboratoire d’Anthropologie et de Psychologie Cliniques, Cognitives et Sociales – LAPCOS, Université Côte D’Azur, Nice, France. Email: fabiana.queiroga@univ-cotedazur.fr https://orcid.org/0000-0002-3811-8202; 3 Social Psychology Department, Research Institute of Personnel Psychology, Organizational Development and Quality of Working Life [Idocal], Universitat de València, València, Spain. Email: nuria.tordera@uv.es https://orcid.org/0000-0002-0379-4657; 4 Bienestar y Capital Social (BYCS), Universidad de Zaragoza, Psychology and Sociology Department, Zaragoza, Spain. Email: mglacruz@unizar.es https://orcid.org/0000-0001-8261-058X

**Keywords:** happy-productive worker, performance, wellbeing, presenteeism, emotions at work, teachers

## Abstract

Numerous reviews have indicated that happiness and performance do not necessarily align in the educational field. Building upon the happy-productive worker thesis (HPWT), but extending its scope, we argue that a person-centered approach may help to explain conflicting results and null findings reported in this literature. To test this proposition, we examined multiple operationalisations of well-being and performance, encompassing both positive and negative valences, and analysed how these operational choices affect the configuration and stability of worker profiles. Data were collected from 1,797 education workers (76.9% women) using validated self-report measures of well-being (positive and negative affect), job performance, and presenteeism. Cluster analyses were conducted using a combination of model-based and *k*-means procedures to identify patterns of synergy and antagonism between well-being and performance. Cluster stability across alternative operationalisations was assessed using the Adjusted Rand Index (ARI). Results support a four-pattern model of relationships between the constructs (happy-productive, unhappy-unproductive, happy-unproductive, and unhappy-productive). However, the distribution of the sample of educational workers across clusters, and the predominance of patterns, differed depending on how performance and well-being were operationalised (positive or negative valences). Cluster stability across operationalisations was low, as indicated by ARI values ranging from .196 to .381. Moreover, some person-related variables (age and sex) accounted for cluster belongingness when well-being was operationalised negatively (negative affect), but not positively (positive affect). These results extend previous research on the relationship between performance and well-being in the HPWT literature. They question the uniqueness of the synergetic hypothesis and highlight the importance of considering the polyhedral nature of both concepts, particularly their positive or negative valence.

## Introduction

The primary objective of work psychology is to enhance the health and well-being of employees while fostering high-performing organisations (Peeters et al., 2014). This goal is underpinned by the belief that happier employees tend to perform better, a proposition known as the happy-productive worker thesis (HPWT). Thus, promotion of well-being and performance has been a perennial goal and the holy grail in management (Wright & Cropanzano, 2007). However, the support for HPWT in the literature is controversial, with re-sults suggesting that the way happiness and performance are operationalised could be influencing the nature of their rela-tionship, and that it is important to study job-related affect (Sender et al., 2020; Wright & Cropanzano, 2007) in educa-tion (Pérez-Nebra et al., 2022). Furthermore, the adoption of a person-centered approach, in contrast to the more common variable-centered approach, has yielded fresh insights into this topic (Cropanzano & Wright, 2001; Gillet et al., 2022). Previous research has identified different patterns of relationship between performance and well-being depending on how these are operationalised (Peiró, Kozusznik, et al., 2019). Notably, job-related affect, encompassing both positive and negative valences, has emerged as a crucial construct for investigation (Sender et al., 2020). Against this background, the present study aims to apply a person-centered approach to the study of the relationship between wellbeing and performance, analysing different patterns of relationships between different operationalisations of those variables in the field of education.

## The happy-productive worker thesis

The HPWT proposes a positive association between wellbeing and performance. However, empirical research reported in international reviews ranges its relationship from null to weak (Cropanzano & Wright, 2001; Daniels & Harris, 2000; García-Buades et al., 2020; Sender et al., 2020), thus showing results that do not confirm the expected relationship between these two variables. Longitudinal studies have supported the association while challenging its unidirectional nature (Magdaleno et al., 2020; Peiró, Tordera, et al., 2019). Moreover, negative associations between these variables have also been observed (García-Buades et al., 2020; PérezNebra et al., 2021). These findings collectively suggest that the HPWT is potentially oversimplified, leaving the relationship between well-being and performance unresolved (Peiró, Kozusznik, et al., 2019).

As such, the relationship between these variables poses critical challenges that can be categorised into four main groups. The primary concern is that the approach in HPWT studies is mostly variable-centered. The second challenge refers to the lack of consideration of the polyhedral nature of both constructs, well-being and performance, and how they are conceptualised and measured (Warr & Nielsen, 2018). The third aspect concerns the bias toward positive operationalisations of performance and well-being in HPWT research, at the expense of other operationalisations and conceptualisations that reflect negative valences (e.g., ill-being and counterproductive performance). Finally, the fourth concern refers to the degree of within-person stability in the patterns of relationship between well-being and performance across different conceptualisations and operationalisations of those constructs and their antecedents. That is, do individuals present the same pattern (e.g., a synergetic happy-productive pattern) under different operationalisations of well-being and performance (e.g., as measured in terms of positive affect and performance, or in terms of negative affect and presenteeism)?

## Beyond the happy-productive worker thesis

The HPWT commonly adopts a variable-centered approach that assumes a synergistic relationship between wellbeing and performance. Synergistic means that the constructs (well-being and performance) are positively related, in the sense that individuals with high scores on one variable tend to have high scores on the other. This type of analysis is variable-centered, as it describes relationships between variables within individuals and has a nomothetic function, like the hypothetical covariate constructs, assuming linearity and investigating averages (Valsiner, 2015).

To advance research in this area, it is necessary to broaden the scope beyond the traditional HPWT framework. One proposed approach involves investigating various patterns of relationships, encompassing both synergistic and antagonistic patterns, across diverse operationalisations of well-being and performance (Peiró, Kozusznik, et al., 2019). This approach can help identify personal and organisational factors associated with these patterns and their long-term sustainability (Tordera et al., 2020). This might also lead to a heuristic understanding of the organisational and personal factors that increase the synergies and reduce the constructs’ antagonisms. This approach considers that well-being and performance are better understood when the relationship is described in terms of patterns instead of linearity.

Previous research adopting this approach has found that the synergetic relationship found between well-being and performance was only a part of the story (Peiró, Kozusznik, et al., 2019). In this sense, by assuming that a happy worker is productive, we are ignoring those who feel differently and making no effort to explain or understand their circumstances.

**Figure 1:**
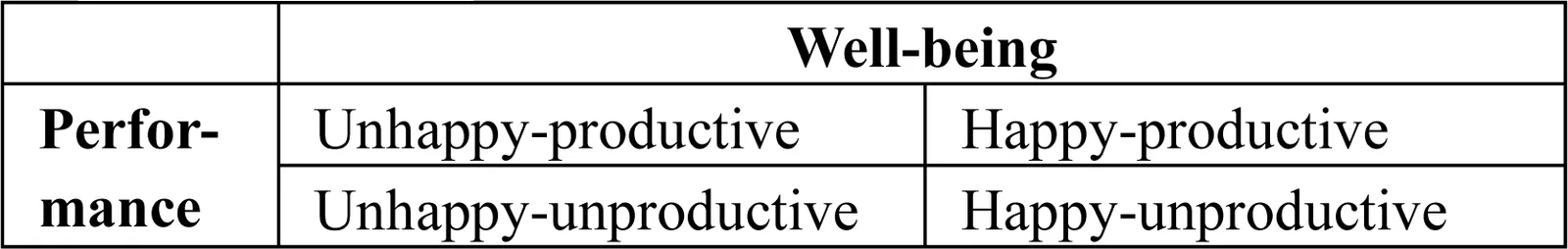
Peiró, Kozusznik et al.’s well-being and performance quadrant relationship proposal

Peiró and colleagues (Peiró, Kozusznik, et al., 2019) proposed four patterns of relationships between well-being and performance. Instead of assuming a linear relationship between well-being and performance, the model integrates anomalous results found in previous studies and includes synergistic and antagonistic relationships between the two variables (Figure 1). As such, these authors propose four patterns: two synergistic (happy-productive and unhappy-unproductive) and two antagonistic (unhappy-productive and happy-unproductive).

Figure 1 shows four standards distributed along two axes – well-being and performance at work – forming four quadrants. The upper quadrants comprise greater performance, while the lower quadrants show less performance. Quadrants to the right comprise higher well-being, and those to the left lower well-being. Empirical support for the four-quadrant model has been found through cluster analysis. Research has consistently identified these patterns across different operationalisations of well-being and performance (Ayala et al., 2017; Latorre et al., 2021). However, there is no consensus in the literature regarding how these patterns are organised, whether their structure varies based on their valence, whether the patterns are stable within-person when different operationalisations of the main constructs are applied, and whether they have other antecedents, thus opening a new avenue of inferences.

In the present paper, we adopt a person-centered approach, thereby broadening the scope of the phenomena by including antagonistic patterns, which reflect a significant negative association between the two variables: a high value for one implies a low value for the other. This alternative approach shifts the focus from the variable to the person and returns the focus to the person in the psychology field (Valsiner, 2015), an aspect that is often overlooked.

A second challenge in this area of research is the positive bias in the conceptualisation and operationalisation of both constructs when considering the relationship from a variable-centered approach (Gutiérrez et al., 2020). Well-being encompasses different dimensions: hedonic (e.g., job satisfaction) and eudaimonic (e.g., personal growth) dimensions related to psychological well-being, as well as context-free dimensions (e.g., life satisfaction) and domain-specific ones (Warr & Nielsen, 2018). However, research in the context of HPWT has mainly operationalised and measured well-being as job satisfaction (Pérez-Nebra et al., 2021; Wright & Cropanzano, 2007); in other words, they focus on one specific aspect of well-being. Furthermore, its relationship with performance (also measured in its positive valence) emphasises positive relations and discards negative or null ones (Peiró et al., 2021).

Nonetheless, research has shown that, depending on the operationalisation of these constructs, the same subjects can be clustered in different quadrants (Peiró, Kozusznik, et al., 2019). Thus, these patterns have been found to be measurement-dependent (Peiró, Kozusznik, et al., 2019), which means they depend on how employee well-being and performance are measured. Thus, in terms of the operationalisation of the constructs, the present study accepts the importance of addressing different indicators of well-being and job performance (Koopmans et al., 2011, 2014; Warr, 2011; Warr & Nielsen, 2018).

Previous research has found that the relationship of wellbeing with other variables may vary depending on the dimensions covered by the definition of well-being; that is, the different dimensions of well-being may have different impacts (Mishra & Venkatesan, 2023), thus leading to mixed results (Sender et al., 2020). The operationalisation of performance also deserves some attention. The dimensions and indicators of performance differ between jobs, but we can consider a heuristic framework including four dimensions: task performance, contextual performance, adaptive (or creative) performance, and counterproductive work behaviour (Koopmans et al., 2011). While the three first dimensions adopt a positive approach to the measurement of performance, counterproductive work behaviour, including absenteeism (not due to illness) and turnover, is considered to have a negative effect on organisations (Campbell & Wiernik, 2015; Collie, 2023; Gillet et al., 2022; Sender et al., 2020; Warr & Nielsen, 2018). Although research recognises the importance of both approaches to understanding performance at work, research is biased toward its positive formulation.

Our study extends the study of performance in relation to well-being, by explicitly including presenteeism ‘when employees are physically present at their jobs [and] they may experience decreased productivity and below-normal work quality’ (Koopman et al., 2002). Productivity loss is evident in the case of presenteeism, but it is less clear when considered under the umbrella of counterproductive work behaviour or the withdrawal construct (Gillet et al., 2022; Harrison & Newman, 2013). Although there is no scientific evidence supporting the hypothesis that presenteeism is necessarily negative (Karanika-Murray & Biron, 2020), we assume that performance loss is harmful and has a negative valence.

Research has shown evidence of the importance of valence in the operationalisation of constructs. For instance, systematic bias has been identified when people assess valence as positive or negative (Sharot & Garrett, 2016). Desirable (i.e., positive) information is integrated into prior beliefs more readily than undesirable (i.e., negative) information, thus resulting in a robust, positively biased evaluation (Sharot & Garrett, 2016). Sharot and Garret (2026) reported that information asymmetry is most likely to be described when it involves motivation and some ambiguity. Thus, workers would maintain a positive view of themselves and their circumstances and ignore negative information. Moreover, reinforcement learning also shows that people learn differently from positive and negative errors and assessments (Sharot & Garrett, 2016). The research conducted by Spreitzer et al. (2009) has yielded similar results. These authors observed that workers’ positive emotions and empowerment increased when they were given information about their strengths and professional development (both in positive and negative valences) (Ayala et al., 2017; Cropanzano & Wright, 2001; García-Buades et al., 2020; Peiró et al., 2021; Peiró, Kozusznik, et al., 2019; Peñalver et al., 2019; Pérez-Nebra et al., 2025; Sender et al., 2020; Spreitzer et al., 2009; Staw, 1986; Wright & Cropanzano, 2007). However, when asked to recall work-related experiences, they frequently referred to negative emotions (Synard & Gazzola, 2017). Evidence in the organisational field indicates that the relationship of negative and positive affect with performance differs when the former is operationalised as either interpersonal citizenship behaviour or interpersonal counterproductive behaviour (Feys et al., 2013), suggesting that the relationship between well-being and performance can vary according to their valence.

## This study

In the present research, we test whether different clusters are identified when using diverse measures of well-being and performance. If the cluster patterns are similar across different measurements, we can assume that the person-centered approach is relevant. In addition, we expand on previous research by adding a visual analysis approach to the cluster analysis.

**Hypothesis 1.** Our first hypothesis is that we will find four patterns, using different measures of well-being and performance valence (H1).

Another relevant question concerns pattern stability within persons. The stability of the *pattern structure* across variables differs from *within-person* stability. Indeed, the relative pattern stability within persons is still less well understood. To sum up, if we measure well-being and performance in different ways, will the same person always be in a cluster with the same pattern? Peiró, Kozusznik et al. (2019) found that although the types of patterns of relationship between constructs were stable across different operationalisations, people were not always stable across these patterns. Depending on how the constructs were operationalised (e.g., selfrated performance vs supervisor-rated), the same individuals were clustered in some cases in one pattern (e.g., happy-productive) and in a different one in others (e.g., unhappy-productive).

In light of the above, we examine the individual stability of the pattern structure across different valences of well-being and performance. Weiss and Cropanzano (1996, p. 28) suggest that negative affect and positive affect ‘seem only to be independent constructs at the high end of the poles’, and there is a long discussion in the literature about valence (Colombetti, 2005) and acquiescence (Schmitt & Stuits, 1985). One problem is related to the idea that emotions can be dichotomised, but valence is often used to ‘refer to how good and bad an emotion feels’ (Colombetti, 2005). This identifies affect valence as the positive and negative character of an emotion experienced, and this is how we will use it, related to work, in the present study. Furthermore, the literature has shown that when the positive and negative valence of the emotion are measured at the same moment, they tend to show higher correlations, which tend to weaken over time (Diener & Emmons, 1984, study 4). Thus, if the constructs of positive and negative affect were not independent, a similar structure would be expected whichever variable pole was measured, in other words, with the same people being clustered in the same pattern. Moreover, we will test whether positive affect and negative affect are opposite or independent constructs, with different perceptions.

**Hypothesis 2.** Our second hypothesis states that, depending on the operationalisation of constructs, educational workers will be grouped in different clusters (i.e., workers are not stable between clusters) (H2).

Peiró, Kozusznik, and colleagues (2019) measured both well-being and performance in their positive meaning. The hedonic well-being measure was job satisfaction, a cognitive aspect of hedonic well-being. Our study extends this research by testing an affective hedonic variable across its positive and negative valences. The measurement of affect at work considers emotions or moods experienced while working (Demo & Paschoal, 2016; Fisher, 2010; Weiss & Cropanzano, 1996; Zelenski et al., 2008). The development and use of scales to measure positive and negative affect is not new in the work and organisational field (Fisher, 2010; Price, 1986) and are mainly based on the PANAS (Positive and Negative Affective Scale) (Demo & Paschoal, 2016; Zelenski et al., 2008).

We assess performance using a task and context performance model (Andrade et al., 2020; Sonnentag & Frese, 2002). Although self-rated performance is susceptible to common-method variance, which is influenced by mood, empirical studies do not fully confirm that positive or negative affect present method biases (Keeping & Levy, 2000). Other biases typically associated with self-reported performance include self-serving bias, social desirability, ‘yea-saying’ (Couch & Kenniston, 1960), and positive assessment. In these cases, employees might protect their self-esteem (Andrade et al., 2020; Sharot & Garrett, 2016); moreover, selfdescriptions can also be positively interpreted because they do not lack adequate knowledge and, in general, less evidence is produced in the relationship between self-rated performance and well-being variables (Warr & Nielsen, 2018). Performance ratings by other people can be problematic, because observers may lack adequate knowledge or because target behaviours depend on unobservable mental processes; furthermore, self-descriptions might err towards positive assessment.

Finally, another concern is whether socio-professional variables or occupational groups promote more sustainable relationships at work (de Jonge & Peeters, 2019; Gordon & Schnall, 2009; Peiró et al., 2014, 2015). Some studies have addressed variables based on personal and work antecedents (Peiró, Kozusznik, et al., 2019). For instance, sex, age and seniority at work have been found to increase the probability of being in the happy-productive worker pattern (Bermejo Toro & Prieto Ursúa, 2014; Warr, 2007; Yin et al., 2018); thus, well-being and positive performance predominate in older men with work experience. These demographic characteristics of employees may also explain life experience and the social units in which people live and work. Additionally, description of socio-demographic characteristics is interesting for person-centered research, and the inclusion of work characteristics is essential to map and facilitate interventions. We investigate these relationships in an exploratory manner based on previous research that shows one or more differences according to the variables measured, most of which are based on well-being.

**Hypothesis 3.** Our third hypothesis is that socio-professional variables will increase the probability of being in different patterns (H3).

To test patterns among variables using different valence measures of well-being and performance, we conducted a survey in the field of education in Brazil, assuming that working in this field entails high emotional demands (Yin et al., 2018). This study contributes to the literature (1) by providing evidence for a pattern structure between well-being and performance; (2) by testing this structure using different operationalizations of performance and well-being, attending to their positive or negative valences; and finally (3) by offering exploratory relationships between socio-professional variables and the patterns found. This study also expands research in the field of education.

## Method

### Participants

The inclusion criteria for this study were: being an employee of the Secretariat of Education of the Federal District (Brazil) and completing the entire questionnaire. Thus, 1,797 employees met the criteria, with a mean age of 44.56 (SD = 7.89); 76.9% were female. Despite the extensive participation of teachers (78.9%), the sample also included other service workers (concierge, cleaner, cook; 8.6%), technicians (nutritionist, psychologist, engineer, doctor, and nurse; 6.2%), and monitors (2.2%). For this sample, we used only online answers.

### Measures

#### Well-being measures

**Positive and negative affect** were measured with a reduced version of the Well-Being at Work Scale *[Escala de Percepção de Bem-Estar no Trabalho] –* EBET (Demo & Paschoal, 2016), including only 9 items corresponding to two of the three original subscales. This scale originally comprised 30 items and three factors, two referring to hedonic well-being (positive affect and negative affect) and one to eudaimonic well-being (personal fulfilment). The reduction was based on the highest factor loadings in each factor, distributed as follows: Positive Affect (4 items, Cronbach’s alpha = .94; omega = .95; e.g., ‘my work made me feel happy’) and Negative Affect (5 items, Cronbach’s alpha = .92; omega = .92; e.g., ‘my work made me feel distressed’). A five-point agreement scale was presented to answer the items. The adjustment indices were appropriate and confirmed the two factors used in this study (χ^*2*^/df = 6.38; CFI = 0.99; TLI = 0.99; NNFI = 0.99; RMSEA = .05; SRMR = .03).

#### Performance measures

**Counterproductive Performance – Presenteeism.** A questionnaire comprising the Stanford Presenteeism Scale (SPS-6). The original version of this instrument was developed and validated by Koopman et. al (Koopman et al., 2002) and was first translated into Portuguese by Ferreira (Ferreira et al., 2010). The instrument was answered on a scale of 1 (complete disagreement) to 5 (complete agreement). There was a ‘no applicable’ option for persons who had never felt presenteeism. The scale comprised two factors: Avoiding Distraction (3 items, alpha = .80; e.g. ‘Because of my health problem, the stress of my job was much harder to handle’) and Completed Work (3 items, alpha = .75; e.g. ‘Despite my health problem, I was able to finish hard tasks at work’). To compose a general indicator of presenteeism, a second-order factor structure was tested (χ^*2*^/df = 49.57; CFI = 0.97; TLI = 0.93; NNFI = 0.93; RMSEA = .17; SRMR = .09). However, because the fit indices indicated poor model adjustments, we followed recommendations from the literature to consider the SPS-6 factors independently (Bezzina et al., 2023). The completed work factor was selected because it showed a stronger negative association with job performance and was theoretically more antagonistic to the alternative performance indicator used in the cluster analyses.

**Self-Assessment Scale of Job Performance.** A singlefactor, self-reported scale containing 10 adapted and reduced job performance items (Cronbach’s alpha = .92; omega = .92; e.g., ‘I take initiatives to improve my results at work’) (Andrade et al., 2020), originally in Portuguese, answered on a 5-point frequency scale. It contains performance items related to task and context. The adjustment indices were acceptable (χ^*2*^/df = 19.29; CFI = 0.99; TLI = 0.99; NNFI = 0.99; RMSEA = .10; SRMR = .05). Although the RMSEA indicated suboptimal fit, other fit indices consistently supported the adequacy of the one-factor model. High CFI and TLI values, together with a low SRMR, suggest that the model adequately captures the underlying construct.

#### Socio-demographic characteristics

We identified the following employee demographic variables. Personal variables were age and sex. Work variables were: years within the organisation (15.78; SD = 7.95); years in the profession (Seniority) (18.89; SD = 7.93); and educational level. Educational level was measured as follows: doctoral degree (1.1%), master of research [master of advanced studies] (7.0%), professional master’s degree [i.e., specialisation] (61.3%), undergrad (26.0%), high school (4.5%), and primary school (0.1%).

### Procedure

Data were collected solely online, with informed consent required. For this data collection, questionnaires were sent by the responsible body to the e-mails registered at the institution.

### Ethical aspects

Prior to starting the study, ethical approval was obtained from the national Brazilian research centre that supported this study (CAAE: 53743316.2.0000.0023). During data collection, international ethical recommendations were followed (the general principles for research involving human subjects from the Declaration of Helsinki, available online: https://www.wma.net/what-we-do/medical-ethics/declaration-of-helsinki/). Initially, the research objectives were explained and respondents were assured that participation was completely voluntary and that they were free to withdraw from the survey at any time if they thought it necessary. We also provided a contact to offer support in case participants felt uncomfortable. All information was treated anonymously and kept confidential throughout the entire process.

### Data Analysis

#### Preliminary analysis

We used listwise deletion of missing data. Hence, 1,797 employees completed the questionnaire in its entirety. In the presenteeism questionnaire, when a respondent answered ‘not applicable’, this was treated as a missing value in order to have a homogeneous sample of all subjects with presenteeism issues. Assumptions and fits were observed. We applied the DWLS estimation method for all CFAs. Well-being at work and presenteeism scales exhibited no normality issues and an acceptable CFA adjustment (Rosseel, 2014) and reliabilities (Jorgensen et al., 2018). However, the job performance variable showed non-normal distribution. Two outliers were identified based on extreme values (interquartile range criterion) and removed; this procedure did not affect CFA results. We also searched for multivariate outliers applying Mahalanobis distance and no participant was identified. All scales ranged from 1 (minimum) to 5 (maximum), and we computed the scale by the average of the items.

#### Cluster analysis

To test the four-pattern hypothesis (H1), we performed cluster analysis using ROP-R free software (Vargha & Bánsági, 2022; Vargha & Grezsa, 2024) with the R (version 4.5.2). All variables were measured on the same scale; however, standardisation was applied to facilitate the identification of ’above’ or ’below’ average profiles. Given the high skewness in performance variables, *Z*-scoring enables clearer comparisons across distinct domains. Model-Based Cluster Analysis (MBCA) (Gergely & Vargha, 2021) and *k*-means were applied (Vargha et al., 2015). Cluster solutions were evaluated based on some quality coefficients (QCs) (Vargha et al., 2016; Vargha & Bergman, 2019), the optimum number of clusters (OptK) and graphical inspection.

Initial MBCA solutions yielded a wide range of optimal number of clusters (*k* = 10, 11, 24 and 25), which were considered inadequate due to low interpretability, high variability, and suboptimal cohesion indices. Therefore, MBCA was performed in a second run by restricting the number of clusters to *k* = 5.

To test cluster membership hypothesis (H2), we assessed the stability of individuals’ classification across alternative clustering solutions using the Adjusted Rand Index (ARI) (Dalmaijer et al., 2022). ARI values were computed in R (package mclust) by comparing cluster assignments (Scrucca et al., 2016). Additionally, we computed the cell matching ratios (CMRs) for the corresponding 4 cluster pairs. To compute it, we used the Exacon module in the Patternoriented analysis menu point of ROPstat (Vargha et al., 2015, 2024).

#### Regression analysis

The number of cases per cluster is balanced, as not all participants include themselves in the happy-productive cluster. This diversity of cases favours the use of multinomial logistic regression. To conduct the regression analysis, to test H3, we correlated the antecedent variables and, as all correlations were above .70, we could consider the non-additivity of the data. We performed multinomial logit regression using the mlogit package (Croissant, 2019), with the happy-productive synergic cluster as the reference group.

### Results

A correlation analysis was conducted between measures of well-being and performance, which produced values below .30 (see Table 1). The results indicate low linear relationships, which allows us to perform the cluster analysis (Wei et al., 2017). Two different methods were applied to identify the clusters. Table 2 compares the MBCA solution and a theoretically hypothesized 4-cluster solution (using *k*-means – 4 clusters, Hartigan-Wong algorithm), adding information about WSS and Silhouette plots.

**Table 1:** Means, standard deviations and correlations between well-being and performance variables (Cronbach’s alpha values are reported along the diagonal in parentheses).

Variable	Mean	SD	Median	Skewness	Kurtosis	1	2	3	4
1. Negative affect	2.96	1.09	3.00	0.146	-0.785	(0.92)	-	-	-
2. Positive affect	2.67	1.11	3.00	-0.100	-0.840	-.51	(0.94)	-	-
3. Counterproductive Performance	2.46	1.12	2.33	0.654	-0.292	.16	-.25	(0.80)	-
4. Job Performance	4.37	0.57	4.50	-1.00	1.113	-.15	.28	-.23	(0.92)

*Note*. All correlations were significant at *p*<.01. Variables range between 1 (minimum) and 5 (maximum).

**Table 2:** Comparing MBCA (k=5) and k-means 4-cluster solutions (the theoretical proposition).

Model-based (MBCA)	*k*-means - 4 clusters tested, number of clusters based on EESS%	General Evaluation
Negative affect - Counterproductive Performance - Completed work		

5 clusters, VEI EESS% = 52.256 HCmean = 0.96 XBmod = -0.55	EESS% = 69.68 HCmean = 0.61 XBmod = 0.75 OptK= *k*2=0.38; *k*3=0.60; *k*4=0.70; *k*5=0.77 WSS^1^ Scree plot shows between 4 and 5 clusters Silhouette^1^ suggested 10 clusters	The MBCA 5-cluster solution yields an unacceptable negative XBmod and lower EESS% and higher HCmean than the *k*-means solution.

Negative affect – Job Performance		

5 clusters, VEE EESS% = 53.909 HCmean = 0.92 XBmod = -0.023	EESS% = 70.41 HCmean = 0.59 XBmod = 0.74 OptK= *k*2=0.38; *k*3=0.60; *k*4=0.70; *k*5=0.76 WSS^1^ Scree plot shows between 4 and 5 clusters Silhouette^1^ suggested 2 clusters	The MBCA 5-cluster solution presents unacceptable negative XBmod and lower EESS% and higher HCmean than the *k*-means solution.
Positive affect – Counterproductive Performance - Completed work		
5 clusters, EVE EESS% = 44.492 HCmean = 1.11 XBmod = -0.79	EESS% = 70.44 HCmean = 0.59 XBmod = 0.74 OptK= *k*2=0.42; *k*3=0.61; *k*4=0.70; *k*5=0.76 WSS^1^ Scree plot shows 4-5 clusters as the best solution Silhouette^1^ suggested 5 clusters	The MBCA 5-cluster solution shows an unacceptable negative XBmod and lower EESS% and higher HCmean than the *k*-means solution.

Positive affect – Job Performance		

5 clusters, EVI EESS% = 68.92 HCmean = 0.62 XBmod = 0.57	EESS% = 71.82 HCmeanS = 0.57 XBmod = 0.77 OptK= *k*2=0.42; *k*3=0.62; *k*4=0.72; *k*5=0.77 WSS^1^ Scree plot shows 3-4 clusters as the best solution Silhouette^1^ suggested 2 clusters	The MBCA 5-cluster solution presents a lower EESS%, higher HCmean and lower XBmod compared to the *k*-means solution.

Notes:

1 Generated by agglomerative hierarchical cluster analysis. Vargha and Grezsa (2024) criteria = EESS% >70 (in Vargha and Bergman, 2019 is >65); HCmean <0.50; XBmod >0.50. OptK = partial results for obtaining optimal number of clusters based on EESS% (Between-cluster sum of squares/Total sum of squares); criteria = elbow method.

**Figure 2:**
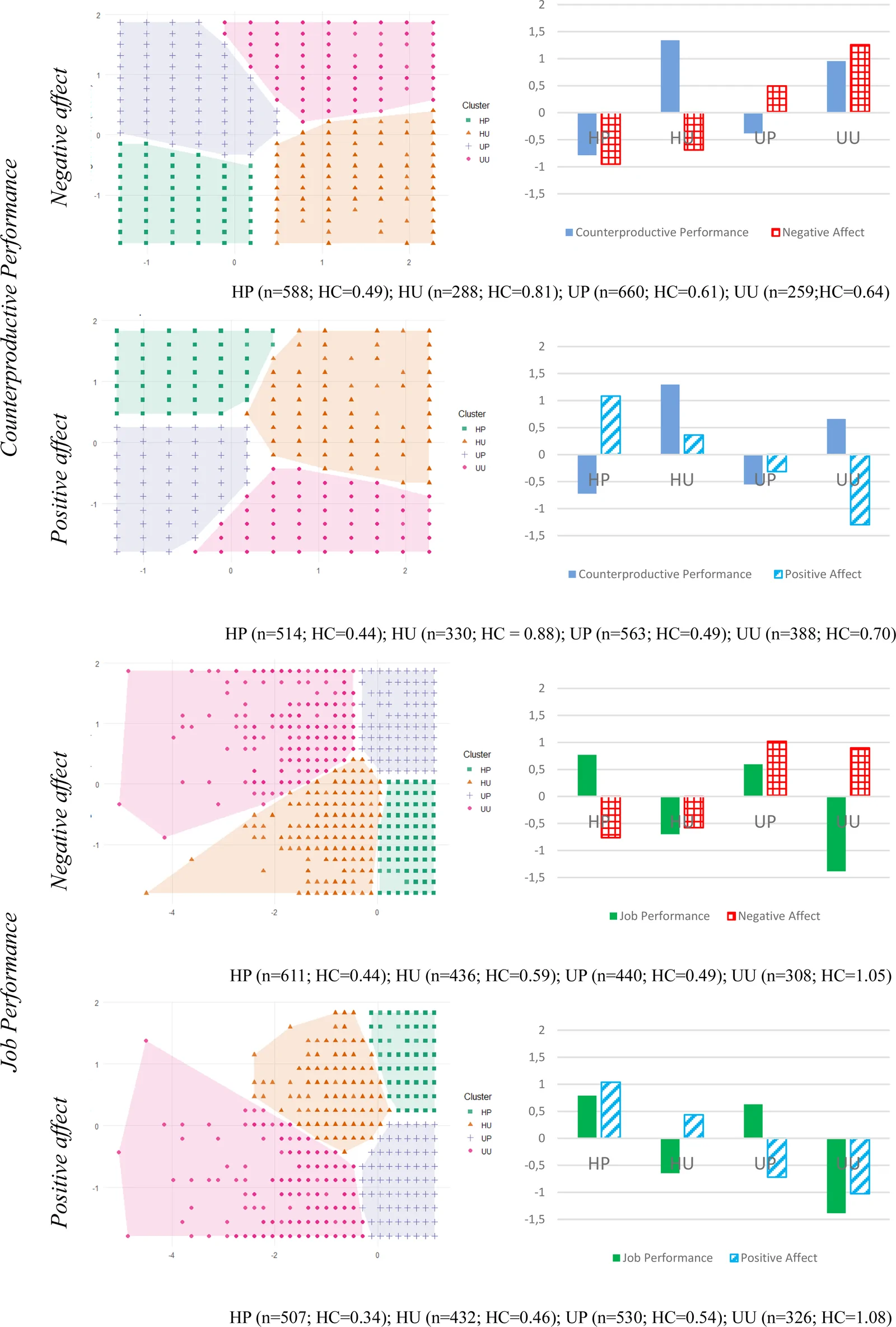
Patterns of the 4-cluster k-means solutions of well-being and performance.

After testing the different solutions and measures for the cluster structure, we concluded that the *k*-means 4-cluster solution is the best option, because four is the smallest number of clusters for each set of variables for which EESS% first reaches 70%. The patterns of the 4-cluster *k*-means solutions of each pair of well-being and performance variables can be seen in Figure 2.

Given that the 4-cluster solution was considered the most adequate, H1 is supported. Figure 2 shows the four-cluster arrangement for the variables. Another result concerns the size of each cluster (as shown in Figure 2), indicating that differences emerge depending on how variables are operationalised. When performance is operationalised as self-reported job performance, most participants were clustered in the happy-productive cluster, followed by slightly lower numbers of subjects in the antagonistic clusters (happy-unproductive and unhappy-productive).

To test cluster belonging (H2), we combined each pair of variable valences (Table 3), i.e., positive and negative affect and positive and negative performance. Table 3 shows that the highest similarity between clustering solutions was observed for the reference variable job performance. When this reference variable was kept constant and only the valence used to measure well-being was changed, the cluster arrangement remained more stable (ARI = .381) compared to the other configurations. That is, the affective core of wellbeing appears to be more robust to differences in valence. CMR analysis suggests the same; the corresponding clusterpair values showed partial agreement (.60-.75) for affective variables. Although more stable than the other solutions, the within-person similarity between clusters remains low, providing support for Hypothesis 2.

**Table 3:** Summary of similarity measures obtained by Adjusted Rand Index – ARI and Cell Matching Ratios - CMR between the same profiles

Reference Variable	Valence Comparison	ARI	Cell Matching Ratios			

			HP	HU	UP	UU
Counterproductive performance	Negative vs Positive affect	.300	0.29	<.2	0.27	<.2
Job performance	Negative vs Positive affect	.381	.71	.70	.63	.70
Positive affect	Job Performance vs Counterproductive Performance	.251	.71	<.2	<.2	.23
Negative affect	Job Performance vs Counterproductive Performance	.196	<.2	.39	.45	<.2

When performance was measured through counterproductive behaviour, the operationalisation of well-being became more relevant for cluster formation (ARI = .284; CMR ranged below .30). Even so, agreement between clustering solutions remained higher than when the valence of the performance indicator was changed. When positive well-being was kept as the reference, changing the performance indicator led to substantial reclassifications (ARI = .249; CMR ranged mostly below .30); however, it is important to notice that the Happy-Productive cluster shows partial agreement. When well-being was defined in terms of negative valence, this classification became even more unstable (ARI = .196; CMR ranged below .50).

Finally, multinomial logistic regression identified four predictors of cluster membership (H3). The pseudo-*R^2^* (McFadden) and its β estimation are described in the tables (Tables 4 and 5). Table 4 presents results between well-being and counterproductive performance (i.e., Completed Work).

**Table 4:** Multinomial logistic regression analysis between well-being and counterproductive performance

	Model 1 Negative Affect			Model 2 Positive Affect		
McFadden R^2^	.015			.004		
χ^2^ (15*df*)	69.29***			19.61		
Cluster	H-U	U-P	U-U	H-U	U-P	U-U
Age	-.012	-.032***	-.074***	-.010	.001	-.019
Sex (1 male 2 female)	-.057	.333**	-.105	-.287	-.076	.026
Years within the organisation	-.001	.011	-.005	.003	.012	-.005
Seniority	-.006	-.001	.022	-.016	-.018	-.001
Educational level	-.157	-.008	.137	-.014	.037	.117

*Note*. Reference cluster is happy-productive.

* p<.05,

** p<.01

*** p<.001

**Table 5:** Multinomial logistic regression analysis between well-being and job performance

	Model 3 Negative Affect			Model 4 Positive Affect		
McFadden R2	.013			.005		
χ^2^ (15*df*)	61.32***			22.18		
Cluster	H-U	U-P	U-U	H-U	U-P	U-U
Age	-.013	-.046***	-.047***	-.023	-.011	-.016
Sex (1 male 2 female)	-.083	.268	.163	-.033	.160	.051
Years within the organisation	.007	.006	.009	.018	.014	.014
Seniority	-.011	.003	-.013	-.017	-.016	-.025
Educational level	-.163	.099	.022	-.009	.146	-.071

*Note*. Reference cluster is happy-productive.

* p<.05,

** p<.01

*** p<.001

In the negative valence of performance (counterproductive) and negative well-being, only two of the professional variables show some differences across the patterns. For older employees, the odds of presenting the happy-productive pattern were higher than the unhappy-productive and the unhappy-unproductive patterns. Similarly, for male employees, the odds of presenting the happy-productive pattern were higher than for the unhappy-productive pattern. However, when measured in terms of positive valence (i.e., positive affect), no differences were found. Table 5 presents the results for well-being and job performance.

Neither did professional variables show differences across patterns of performance and positive affect. In the case of performance and negative affect patterns, older workers were more likely to be in the happy-productive than in the unhappy-productive or unhappy-unproductive patterns. Sex did not play a significant role when measuring the positive valence of performance.

Thus, in summary, no professional or demographic variables were found to predict patterns of performance and wellbeing when the latter was operationalised as positive affect. Age was found to be a relevant variable when considering patterns between both operationalisations of performance and negative affect. Sex was only found to be relevant for patterns of relationship between counterproductive behaviour and negative affect. Although the predictive power of age and sex variables is significant, their strength is low, thereby only partially supporting H3.

## Discussion

The present study adopts a person-centered approach to analyse different patterns of relationships between performance and well-being in a sample of workers in the educational sector. We extend previous research challenging the HPWT by considering positive and negative conceptualisations and operationalisations of performance and well-being. To achieve this goal, we tested the structure’s stability using different measurements and valences and identified a few predictors of the patterns. Upon analysing the relationship between several constructs, we found support for a similar four-pattern structure of the well-being and performance relationship (Hypothesis 1) across variables. We also found support for within-person variability of the clusters depending on the operationalisation of variables (Hypothesis 2). Partly in line with Hypothesis 3, we also found that sociodemographic variables (age and sex) predict the different patterns weakly and differently.

Although seemingly logical, the HPWT thesis remains challenged (Ayala et al., 2017; Cropanzano & Wright, 2001; Peiró et al., 2021; Peiró, Kozusznik, et al., 2019; Peñalver et al., 2019; Pérez-Nebra et al., 2025; Sender et al., 2020). As such, the proposal to use a person-centered approach appears to show some potential. This study supports the stability of the four-cluster pattern structure (happy-productive, unhappy-unproductive, happy-unproductive, and unhappyproductive; Peiró, Kozusznik et al., 2019) when considering the valence of the well-being and performance constructs and corroborates the view of performance and well-being as multidimensional constructs (Mishra & Venkatesan, 2023). As such, the phenomenon is not limited to particular operationalizations. The findings also suggest that antagonistic (misaligned) clusters are as important, or even more so, than synergic (aligned) ones, since antagonistic clusters comprise the same or a larger percentage of employees as in previous research (Peiró, Kozusznik, et al., 2019).

However, we also found within-person variability. Depending on the operationalisation used, people can fall into one pattern or another, which can lead to incorrect decisions in diagnosis and intervention. Results indicate that the empirical classification of individuals into the clusters is unstable, rather than a measure-dependent. While affective wellbeing showed moderate classification stability across alternative operationalisations, productivity indicators led to substantial reclassification, particularly when behavioural measures were used. This work supports Diener and Emmons’s findings of a certain independence between constructs that are measured using different valences (Diener & Emmons, 1984).

Other evidence supporting the four-pattern results has been published previously (Ayala et al., 2017; Peiró, Kozusznik, et al., 2019), with these authors suggesting different predictors for the patterns. Our results indicate that sex plays a role in the patterns, but in different ways. Thus, the results for sex are not stable and could change according to the context. In this study, male workers were more likely to be in the happier-productive pattern only for counterproductive performance (i.e., the Completed Work dimension of presenteeism). However, this sex difference disappears when job performance is measured. It should be noted that for the context studied here (state-run schools) and the sex roles in which the task is conducted, sex-based differences in the educational environment are inconclusive (Bermejo Toro & Prieto Ursúa, 2014; Collie, 2023). However, the suppression of emotion and the relationship between females and anxiety and depression has been repeatedly highlighted in the literature (Yin et al., 2018), although it is also unsettled (Correia Leal et al., 2023; Ferreira & Martinez, 2012). Other samples may show different patterns, suggesting we need to control for potential moderators.

Seniority and years within the organisation were insignificant for our sample, which converges with other results (Bledow et al., 2013; Collie, 2023; Ferreira & Martinez, 2012). The association between old age and less depression, less emotional exhaustion, and greater well-being is unclear as some previous studies have shown positive findings (Marchand et al., 2013) and others no relationship (Bledow et al., 2013; Collie, 2023; Ferreira & Martinez, 2012). The relationship between age and these patterns needs to be clarified and could be context-dependent. Rincón Uribe (2024) found different clusters of affectivity and job satisfaction during the pandemic in a sample of Latin American workers, demonstrating the importance of considering the context when analysing these relationships. In the field of education, which was studied here, being older may perhaps mean greater knowledge and increased recognition at work.

In summary, several factors can influence whether an employee belongs to one cluster rather than the other. Therefore, new personal or organisational antecedents, as well as reciprocal and developmental relationships, need to be included in new studies. The dynamics of reciprocity and the stabilitychange of cluster belongingness are challenges that need longitudinal approaches to answer.

### Limitations

As is the case with most research in this field, this study only investigates the relationship between well-being and performance at one moment in time. The prolonged membership of non-sustainable clusters and their possible impacts have not been studied. Clearly, further research is needed to examine the long-term effects of belonging to these clusters. Moreover, clarifying the different nuances of the relationship between well-being and performance, how a reciprocal or spiral relationship could be built, and its predictors, constitutes a new avenue for theoretical advancement and research in this field.

Empirical research on self-rated job performance has previously also produced high scores (Andrade et al., 2020; Sharot & Garrett, 2016). The scale used in this study presents some issues in its factorial structure, despite its high reliability. Further studies would benefit from testing performance with other informants. Additionally, in the present research, all employees in this sample exhibit some level of presenteeism. These findings could be interpreted in two ways. The first is a self-serving bias, as the field of education in Brazil receives low ratings in standard evaluations, such as the Programme for International Student Assessment (PISA) and Brazil’s System of Basic Education Assessment (Sistema de avaliação da educação básica – SAEB). Public education, where this research was conducted, is a typical example. Another way to interpret the findings is that employees understand their tasks as not limited to the assessment done, mainly because these state-run schools serve vulnerable groups of children. In this sense, they understand that they are doing a meaningful and essential job for the children and society, that these learning assessments do not identify. Clarification of these differences is another avenue for future research. An attempt to understand the differences between these clusters and the national evaluation could improve the national ratings of student learning.

The respondents were from public schools in Brazil, and although it is not generalizable to the whole world, the sample represents the Global South (mostly women, 40 years old +, presenteeism issues). Differences among school actors are also an issue, since there is different treatment within the school on the part of both the management and the students’ parents themselves. All the school players help in education, each with a different role, and all of them need to be seen. In particular, the monitors who deal with pupils with special needs have a slightly different profile within the school and look after pupils who are also different, and looking after the carer is no small matter.

### Practical implications

An important innovation of this study, with practical implications, is the empirical assessment of cluster patterns instead of aligned variable relationships. The results support previous findings, namely that the happy-productive relationship is not necessarily synergistic, as the antagonistic clusters have a similar size as the synergic ones, and different predictors are found for different clusters. Thus, in terms of human resource management practice, it seems that clusters matter for intervention. One strategy could be mentoring younger staff by older staff, as employees who teach their strategies can generate more sustainable patterns at work.

Another point to note is that the way we measure performance or well-being will lead to different patterns of results. In practical terms, organisations usually measure only positive performance, leaving aside other variables in which age could play a role, therefore favouring older people in this educational context.

### Conclusion

Our results provide challenging new avenues for future empirical research that may incorporate other nuances of the valence-related variables, test their antecedents, and their reciprocity. Furthermore, our findings show that varying valences play a key role in the cluster structure and the antecedents presented. The use of a person-centered approach seems to be a critical step to understanding human behaviour in the work context. The personal and work antecedents presented here align with the field of education. The strategy of applying patterns will enable us to take early, preventive actions to promote sustainable, more person-centered work. Future research could include the person over the longer term, their reciprocal and processual relationship, and how we can promote sustainable positive work as psychologists.

## Data Availability

The data that support the findings of this study are available: Pérez-Nebra, A. R.; Queiroga, F. (2022), “Secretaria de Educação”, Mendeley Data, V1. https://doi.org/10.17632/6dkp97x3m4.1

## Action editor

Lars-Gunnar Lundh served as action editor for this article.

## Open access

This article is distributed under the terms of the Creative Commons Attribution 4.0 International License (http://creativecommons.org/licenses/by/4.0/), which permits unrestricted use, distribution, and reproduction in any medium, provided you give appropriate credit to the original author(s) and the source, provide a link to the Creative Commons license, and indicate if changes were made.
